# Molecular and biological characterization of ϕRs551, a filamentous bacteriophage isolated from a race 3 biovar 2 strain of *Ralstonia solanacearum*

**DOI:** 10.1371/journal.pone.0185034

**Published:** 2017-09-21

**Authors:** Abdelmonim Ali Ahmad, Michael J. Stulberg, John Patrick Mershon, Dimitre S. Mollov, Qi Huang

**Affiliations:** 1 Floral and Nursery Plants Research Unit, United States National Arboretum, U.S. Dept. of Agriculture-Agricultural Research Service, Beltsville, Maryland, United States of America; 2 Department of Plant Pathology, Faculty of Agriculture, Minia University, El-minia, Egypt; 3 National Germplasm Resources Laboratory, U.S. Dept. of Agriculture-Agricultural Research Service, Beltsville, Maryland, United States of America; Oklahoma State University, UNITED STATES

## Abstract

A filamentous bacteriophage, designated ϕRs551, was isolated and purified from the quarantine and select agent phytopathogen *Ralstonia solanacearum* race 3 biovar 2 strain UW551 (phylotype IIB sequevar 1) grown under normal culture conditions. Electron microscopy suggested that ϕRs551 is a member of the family *Inoviridae*, and is about 1200 nm long and 7 nm wide. ϕRs551 has a genome of 7929 nucleotides containing 14 open reading frames, and is the first isolated virion that contains a resolvase (ORF13) and putative type-2 phage repressor (ORF14). Unlike other *R*. *solanacearum* phages isolated from soil, the genome sequence of ϕRs551 is not only 100% identical to its prophage sequence in the deposited genome of *R*. *solanacearum* strain UW551 from which the phage was isolated, but is also surprisingly found with 100% identity in the deposited genomes of 10 other phylotype II sequevar 1 strains of *R*. *solanacearum*. Furthermore, it is homologous to genome RS-09-161, resulting in the identification of a new prophage, designated RSM10, in a *R*. *solanacearum* strain from India. When ORF13 and a core *attP* site of ϕRs551 were either deleted individually or in combination, phage integration was not observed, suggesting that similar to other filamentous *R*. *solanacearum* ϕRSM phages, ϕRs551 relies on its resolvase and the core *att* sequence for site-directed integration into its susceptible *R*. *solanacearum* strain. The integration occurred four hours after phage infection. Infection of a susceptible *R*. *solanacearum* strain RUN302 by ϕRs551 resulted in less fluidal colonies and EPS production, and reduced motilities of the bacterium. Interestingly, infection of RUN302 by ϕRs551 also resulted in reduced virulence, rather than enhanced or loss of virulence caused by other ϕRSM phages. Study of bacteriophages of *R*. *solanacearum* would contribute to a better understanding of the phage-bacterium-environment interactions in order to develop integrated management strategies to combat *R*. *solanacearum*.

## Introduction

*Ralstonia solanacearum* causes bacterial wilt, a soil-borne vascular disease that is arguably one of the most economically important bacterial diseases in the world. It attacks over 450 plant species and limits the production of such economically important crops as tomato, tobacco, potato and banana [1]. The race 3 biovar 2 (r3b2) strains (phylotype IIB, sequevars 1 and 2) of *R*. *solanacearum* causing devastating potato brown rot are quarantine pathogens in many countries and are also select agents in the United States [2].

*R*. *solanacearum* normally enters plants from soil through wounds in the roots and then multiplies in the xylem vessels and spreads through the plants’ vascular system. Control of *R*. *solanacearum* depends mainly on quarantines, use of pathogen-free propagating materials and eradication. The use of resistant cultivars, when available, and proper rotation or fallow has also been used with limited success due to the pathogen’s wide host range, broad distribution, great variability and ability to survive in soil and water [3,4]. Recently, the potential of using bacteriophages to control *R*. *solanacearum* has also been explored [5–7].

At present, a wide range of bacteriophages specifically infecting *R*. *solanacearum* have been isolated from soil of crop fields and include: filamentous phages ϕRSM1, ϕRSS1 [8], RS603 [9] and PE226 [10] of the family *Inoviridae*, icosahedral phages of the family *Myoviridae* [5,8], and lytic phages of the families *Podoviridae* [5,11,12], and *Siphoviridae* [13]. All of the *R*. *solanacearum* phages reported so far were isolated from soil in Japan, with the exceptions being the phages studied by Bhunchoth et al. [5] and Murugaiyan et al. [10] that were isolated from soil in Thailand and Korea, respectively.

The filamentous Ff-type phages ϕRSS1 and ϕRSM1 have been studied in detail, including morphological and genomic characterization [8,14], integration mechanism [15,16], potential as a vector/expression vector [14,17], and effect on virulence of their *R*. *solanacearum* host strains [8,18,19]. While the phage ϕRSS1 was found to enhance virulence [18], infection by the phage ϕRSM1 resulted in loss of virulence of *R*. *solanacearum* on tomato plants [19], suggesting different effects caused by the two filamentous phages.

In addition to the *R*. *solanacearum* phages isolated from the soil, Askora et al. [15,20] identified seven ϕRSM-like prophage sequences in the deposited genomes of *R*. *solanacearum* and *R*. *pickettii* with different evolutionary origins, including prophage RSM3 in MAFF7390139 (Asia), RSM4 in UW551 (Americas), RSM5 in IPO1609 (Americas), RSM6 in CMR15 (Africa), RSM7 in Y45 (Asia), RSM8 in *R*. *syzygii* R24 (Indonesia) and RSM9 (*R*. *pickettii* 12J). Askora et al. [15] converted one of the prophages, RSM3, into an infectious phage by PCR and cloning. In addition, open reading frames encoding for different types of putative phage repressors were identified in RSM phages and prophages [20] and RSM-related phage ϕRS603 [9], which may be important for establishing and maintaining the phage lysogenic state and host immunity [20], and for the effect on virulence of *R*. *solanacearum* [19].

In unrelated research, the presence of a bacteriophage was suspected in the supernatant of *R*. *solanacearum* r3b2 strain UW551. We therefore made attempts to isolate and purify the phage, characterize its morphology and genome, compare to other *R*. *solanacearum* phages and prophages, determine its integration mechanism and reveal its biological effect on its susceptible host strain of *R*. *solanacearum*.

## Materials and methods

### Bacterial strains and growth conditions

Strains of *R*. *solanacearum* used in this study are listed in Table 1. To grow *R*. *solanacearum* or phage-infected susceptible *R*. *solanacearum* strain RUN302, the bacterium was freshly streaked from a frozen stock onto triphenyltetrazolium chloride plates [21]. Then, a single colony was picked and grown overnight in casamino acid peptone glucose (CPG) broth [22] at 28°C with shaking. To prepare *R*. *solanacearum* inocula, appropriate concentrations of the bacterial suspensions were made in sterile water using OD_600_ as an initial measurement of cell density. Final inoculum cell density was confirmed by ten-fold serial dilution plating.

**Table 1:** Susceptibility of *R*. *solanacearum* strains to ϕRs551.

*R*. *solanacearum* Strain (phylotype/sequevar)	Origin	Source	Susceptibility to ϕRs551^a^
*r3b2 strains*			
UW551 (IIB/1)	Kenya	C. Allen, USA	**-**
UW501 (II)	Indonesia	C. Allen, USA	**-**
UW276 (II)	Mexico	C. Allen, USA	**-**
RUN628 (IIB/2)	Colombia	P. Prior, France	**+**
4155 (IIB/1)		NCPPB, UK	**+**
RUN160 (IIB/1)	Reunion	P. Prior, France	**-**
RUN440 (IIB/1)	Uruguay	P. Prior, France	**-**
IPO1609 (IIB/1)	Netherlands	P. Prior, France	**-**
RUN256 (IIB/1)	Taiwan	P. Prior, France	**-**
*Non-r3b2 strains*			
RUN302 (IIB/4)	Brazil	P. Prior, France	**+**
BDB (IV/10)	Indonesia	P. Prior, France	**-**
P446 (IIB/4)	USA	D. Norman, USA	**-**

^a^Susceptibility of *R*. *solanacearum* strains to ϕRs551 is shown as susceptible (+) or resistant (-).

### Bacteriophage isolation, purification, and characterization

A bacteriophage designated ϕRs551 was isolated and purified directly from an overnight culture of *R*. *solanacearum* strain UW551 grown in CPG broth at 28°C with shaking, and used for further study. A total of 2 liters of the bacterial culture was grown in order to obtain a sufficient amount of phage particles for morphological characterization and for DNA extraction. The overnight cell culture of UW551 was pelleted at 8000 *g* for 15 minutes at 18°C, and the supernatant was filtered through 0.2 μm membrane filters, followed by ultracentrifugation at 109,000 *g* through a 30% sucrose cushion for 2 hours at 10°C. The phage pellet was resuspended in SM buffer containing 50 mM Tris/HCl at pH 7.5, 100 mM NaCl, 10 mM MgSO4, and 0.01% gelatin [23], stored at 4°C in complete darkness, and used for DNA extraction and morphological characterization. To characterize the phage, the purified phage particles were stained with sodium phosphotungstate using the method of Dykstra [24] prior to observation under a Hitachi HT7700 transmission electron microscope. The size of ϕRs551was estimated from 20 phage particles based on scale bars.

### Phage susceptibility test

To determine the host specificity of ϕRs551, the purified phage was subject to the spot test and plaque-forming assay using *R*. *solanacearum* strains in Table 1 as hosts. This was done by mixing 500 μl of *R*. *solanacearum* cells (OD_600_ of 0.2) with 3.5 ml of CPG containing 0.45% agar and layering the mixture on top of a CPG plate containing 1.5% (w/v) agar. After the top layer with *R*. *solanacearum* was hardened, 3 μl of the purified phage suspension was spotted on the double-layered plate, incubated at 28°C for 24 h for the presence or absence of plaques.

### Phage DNA extraction, cloning, sequencing, analysis and characterization

Phage DNA was extracted from purified phage (described above) using a phenol-chloroform method [23]. The near-entire full length circular phage genome (7694 out of 7929 bases) was cloned into the Zero Blunt vector (Life Technologies, Inc.) using primers F-ϕRs551 and R-ϕRs551 (Table 2) designed on the prophage region in UW551 contig0570. The genomic DNA sequence of ϕRs551 was determined by primer walking the cloned 7694-bp genome fragment. The F-ϕRs551 and R-ϕRs551 primer sequences were confirmed by sequencing purified phage DNA, and the remaining 235 bp not cloned into the vector were sequenced directly from purified phage DNA to close the genome. Potential ORFs of ϕRs551 were identified using GeneMarkS [25] or the online program ORFfinder (http://www.ncbi.nlm.nih.gov/orffinder), and were compared with those of other *R*. *solanacearum* related phages by BLASTP. To assign possible functions to the ORFs and to identify putative *att*P region, database searches were performed using FASTA, FASTX, BLASTN and BLASTX programs [26]. To determine the nature of the ϕRs551 genome, approximately 300 to 500 ng of phage genome was subjected to individual enzyme digestions for 30 minutes in a 30-μl reaction volume with 1 unit of DNase I, 3 units of RNase A, 10 units of exonuclease I at 37°C, or 10 units of S1 nuclease at room temperature (Thermo Fisher Scientific, Waltham, MA.).

**Table 2:** List of primer pairs designed and used in this study.

Primer pair	Sequence (5’-3’)	Position in ϕRs551 or *R*. *solanacearum* strain P082 (italicized)	Size of PCR product (bp)	Target/mutant
F-ϕRs551	CTTCGGCGTCTTCAACATCGGACAGGG	3593–3619	7694	97% ϕRs551
R-ϕRs551	ATCACCCCGCTCAGAGAAACGCAATCC	3331–3357		
P1-F	ACTCCGAACGGGGTAACTCTGTTTT	7800–7824	7534	ϕΔ*att*P
P2-R	TAGGGCAGCCAGTACAATCC	7404–7385		
P3-F	AGCCGTATAGCATGCAACCCCTCG	7345–7368	7359	ϕΔ*orf13*
P4-R	CAGCATAACCACCGCTAAACGAACG	6774–6750		
P5-F	GAACTCCTCCCGTGCTTAGGGCGA	7430–7453	7274	ϕΔ*att*P&Δ*orf13*
P4-R	CAGCATAACCACCGCTAAACGAACG	6774–6750		
P6-F	CAAAAGCTGACCATCATCGCCATC	24–47	7929	Full length ϕRs551
P7-R	AGTGTTCGACATGATGGCTCCGA	23–1		
P8-F	*GATTTTGGGTGTGCAAGGAT*	*1171467–1171448*	290	*att*R
P9-R	GAACCTGCTTAGGGCCAAGA	7503–7484		
P10-F	GCTGCCGATACTGCGATGAA	6719–6738	868	*att*L
P11-R	*GCCTGAGAAAGATTGCATCG*	*1171095–1171114*		
P12-F	GTAATGCTTGCGCTGCAC	*857561–857578*	147	*R*. *solanacearum* genome
P13-R	GCGTCTGATCTGCACTTGTC	*857451–857432*		
P14-F	GAGGTCGTCATGGTCGATCC	395–414	600	Partial episomal ϕRs551 or mutant phages
P15-R	CGCCGTGAATCAACATCGAC	994–975		

### Infection of susceptible *R*. *solanacearum* strain RUN302 with ϕRs551

To infect susceptible *R*. *solanacearum* strain RUN302, which did not contain ϕRs551 sequence in its genome before infection, a single-plaque isolate of ϕRs551 was used. Its titer was determined by mixing 100 μl of the serially diluted phage filtrate with 400 μl of 2 x 10^8^ cells of RUN302 for the plaque-forming assay as described above. For propagation and purification, one milliliter of the overnight culture of RUN302 was diluted with 100 ml of fresh CPG in a 500 ml flask. When the culture reached an OD_600_ of 0.2, ϕRs551 was added at a multiplicity of infection (moi) of 0.001–1.0. After further growth for 24-48h at 28°C with shaking, the phage was purified as described above. Extrachromosomal DNA was isolated from phage-infected *R*. *solanacearum* cells by the minipreparation method [27].

### In vitro growth of *R*. *solanacearum* strains

To study the effect of phage infection on the in vitro growth of its susceptible *R*. *solanacearum* strain, ϕRs551-infected and uninfected *R*. *solanacearum* RUN302 strains were grown overnight in 5 ml of CPG broth. One milliliter of the overnight culture (adjusted to OD_600_ of 0.003) was then transferred into a well of a 24-well sterile microplate, and was grown at 28°C with shaking at 200 rpm inside of a Epoch2 microplate reader (BioTek Instruments, Winooski, VT). The absorbance at 600 nm was measured and graphed every three hours over the course of 48 hours. There were three replicates for each time point per strain, and the experiments were repeated three times.

### Construction of phage mutants

To determine the role of a core 13-nucleotide *att*P sequence and ORF13 in the integration of ϕRs551 into *R*. *solanacearum* susceptible strain RUN302, three ϕRs551 mutants were constructed: 1) ϕΔ*att*P missing a 395-bp fragment containing the core *att*P sequence, 2) ϕΔ*orf13* with a 570-bp deletion including almost all the ORF 13 sequence (190 out of 196 amino acids), and 3) ϕΔ*att*P-Δ*orf13* missing a 655-bp region containing both the *att*P and 582 of the 591 nucleotides of the ORF 13 gene sequences. The mutants were generated by PCR amplification of shortened phage regions using primer pairs (Table 2) designed based on the sequence of ϕRs551. As a positive control, the full length ϕRs551 was also amplified by PCR using primer pair P6-F/P7-R (Table 2). The PCR product was then purified and circularized with a T4 DNA ligase (Promega Corp., USA). The resulting circularized phage mutant or full length phage DNA was electroporated into competent cells of the wild type strain RUN302 using a MicroPulser Electroporator (Bio-Rad, Hercules, CA) with settings of 1.5 KV and 4 milliseconds. This was done by mixing 45 μl of the competent cells with 2μl of the circularized DNA (approximately 40 ng) and placing the mixture in a 0.1 cm—gap electroporation cuvette (Bio-Rad) pre-chilled on ice. The mixture in the cuvette was washed immediately after electroporation with 1 ml of CPG broth and incubated at 28°C for at least 7 hours. Electro-competent cells were prepared by growing overnight cultures of *R*. *solanacearum* to an OD_600_ between 0.4 and 0.7, followed by centrifugation at 4182 x *g* for 10 minutes and four successive washes at 18°C in 1, 0.5, 0.1 and 0.01 volumes of 10% glycerol [28]. The knocked-out regions in the mutants was confirmed by PCR using primer pairs located within the sequences of the mutated regions that showed a lack of any amplified products.

### Determination of the phage integration mechanism

To determine the right and left integration flanking regions (*att*R and *att*L) in *R*. *solanacearum* Run302 after ϕRs551 integrated into the bacterial genome at a potential *att*B site, total genomic DNA was extracted from infected RUN302 cells using the DNeasy Blood and Tissue kit (Qiagen, Inc.), and amplified using primer pairs P8/P9 and P10/P11 (Table 2). Since the genome sequence of RUN302 is not available, the P8 and P11 primers targeting *R*. *solanacearum* RUN302 sequences were designed based on the potential *dif* (*att*B) sequence identified in the fully sequenced *R*. *solanacearum* strain P082 [29], a closely related strain which is also a biovar 1, phylotype IIB, sequevar 4 strain like RUN302. The PCR products were purified from agarose gels with the QIAquick gel extraction kit (Qiagen, Inc.) and sequenced commercially.

To determine the time needed for ϕRs551 to integrate into the RUN302 genome, total genomic DNA of ϕRs551-infected RUN302 was extracted every hour for seven hours for PCR determination of the *att*R and *att*L regions as described above. As a control, the DNA was also amplified by PCR using primers P12 and P13, designed based on *R*. *solanacearum* strain P082, to detect the host bacterial genome.

To determine whether phage integration was affected by mutations in *att*P and orf13, total genomic DNA from RUN302 infected by each of the three phage mutants constructed above was purified and subjected to PCR using the P8/P9 and P10/P11primers pairs. The primer pair P14/P15 was also used to determine whether circular episomal forms of ϕRs551 were produced. Uninfected wild type RUN302 genomic DNA was used as a control in all the experiments.

### PCR conditions

PCR using primer pairs P8/P9, P10/P11, P12/P13 or P14/P15 was performed in a 20-μl volume containing 1x GoTaq Green Master Mix (Promega, Madison, WI) and 5 pmol of each primer. Twenty nanograms of DNA was added to the reaction mixture. PCR conditions were 1 cycle of 4 min at 94°C, 30 cycles of 1 min at 94°C, 1 min at 60°C, and 1 min at 72°C, with a final extension of 10 min at 72°C. For PCR using primer pairs of P1/P2, P3/P4 or P5/P4, 1x KAPA HiFi HotStart ReadyMix (KAPA Biosystems, Boston, MA) was used and the PCR conditions were 1 cycle of 3 min at 95°C, 30 cycles of 20 sec at 98°C, 15 sec at 62°C, and 2.5 min at 72°C, with a final extension of 8 min at 72°C. For PCR using primer pair P6/P7, the PCR conditions were the same as P1/P2, P3/P4 or P5/P4, except that the annealing temperature was 59°C. Five microliters of each PCR product was mixed and visualized by electrophoresis in 1.0% agarose gels stained with GelRed (Phenix Research Products, Candler, NC).

### Extracellular polysaccharide (EPS) assay

EPS in bacterial culture supernatants was determined quantitatively using a modified method of Ahmad et al. [30] and Jeong et al. [31]. Briefly, bacterial strains were grown in CPG medium for 72h at 28°C with shaking at 200 rpm. The optical density (OD) at 600 nm was measured for each bacterial suspension and adjusted to the same OD_600_ with liquid CPG. Ten milliliters of each strain were then centrifuged at 5000 × g for 20 min at 4°C, and the supernatant was collected and passed through 0.22 um membrane. The filtrate was mixed with 4 volumes of acetone and a final concentration of 20 mM NaCl, and the mixture was kept overnight at 4°C. To determine the dry weight of EPS, the precipitated EPS was collected by centrifugation and dried at 55°C prior to measurement. Two replicates were used for each strain and the experiment was repeated three times.

### Motility assay

Swimming and swarming motilities of the wild type strain RUN302 of *R*. *solanacearum* were examined on CPG containing 0.3% and 0.7% (w/v) agar, respectively, and compared to those of ϕRs551-infected RUN302 strain. Overnight cultures of the RUN302 strains in CPG were centrifuged at 8000 *g* for 2 min at 4°C, washed twice with sterile water, and resuspended in water to an OD_600_ of 0.1, approximately 10^8^ cells per milliliter. Three microliters of the suspension were spotted in the center on an agar plate containing 20 ml of CPG and appropriate amount of agar, and incubated at 28°C. The plates were photographed 5 days after incubation. For twitching motility assays, 3 μl of the suspension were spotted in the center on a minimal medium plate [19], incubated for 5 days at 28°C. Twitching motility was then visualized by placing the plate without its lid on the stage of a light microscope (Carl Zeiss Microscope GmbH, Germany) under 40× magnification, photographed using OMAX digital camera (OMAXmicroscope.com), and saved to OMAX ToupView software in gray scale as uncompressed TIFF files. Colonies with twitching motility were characterized by the formation of corrugated trajectories around the colonies.

### Virulence assay

Seed grown tomato plants (*Lycopersicom esculentum* Mill. cv. ‘bonnie best’) were transplanted approximately 7–10 days after germination, and inoculated two to seven days after transplanting, using the soil drenching method to mimic the natural infection process and maintained as described before [32], except that 50 ml suspensions with 5 x 10^7^ cells of *R*. *solanacearum* were poured into each pot. Water was used as a negative control. Inoculated plants were rated daily for three weeks using a disease index of 0 to 4 [33]. There were 10 plants per treatment and the experiment was repeated three times.

### Statistical analysis

The means of growth rate, EPS dry weight, and disease index between the wild-type and ϕRs551-infected *R*. *solanacearum* RUN302 strains were analyzed for significant differences using the t test in Microsoft Excel.

### Nucleotide sequence accession numbers

The genome sequence of ϕRs551 has been submitted to GenBank and given accession no. KX179905. The accession numbers for M13 phage and for other *R*. *solanacearum* filamentous phage sequences used in this study are: M13, NC_003287; RSM3, AB434711; and RS603, AB937974.

## Results

### Morphology and host specificity of ϕRs551

**Fig 1:**
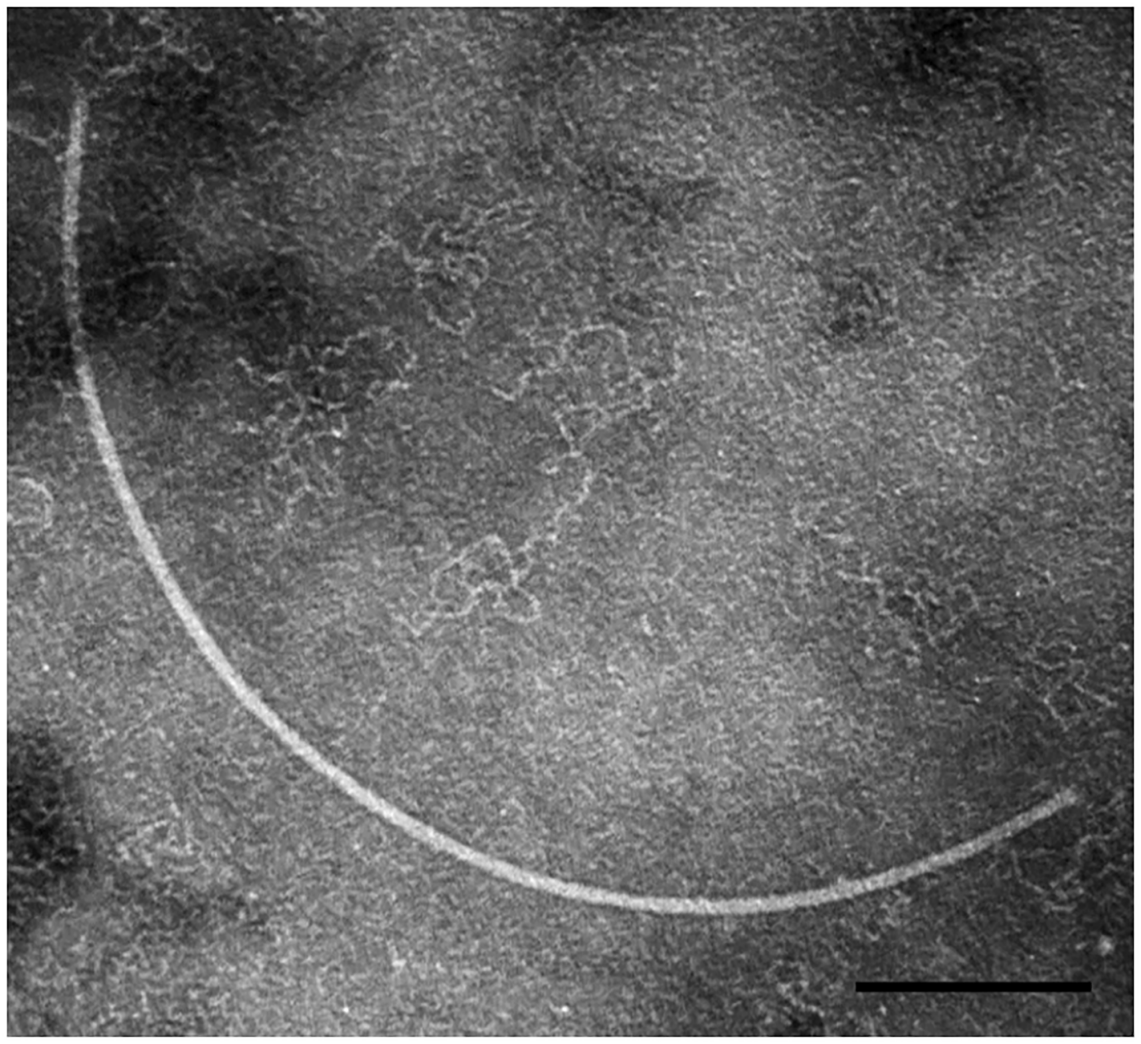
Electron micrograph of the purified ϕRs551 virions. The black scale bar represents 200 nm.

When the supernatant of the overnight culture of *R*. *solanacearum* strain UW551 grown under normal growth conditions was purified, a flexible filamentous particle, resembling the members of *Inoviridae*, was observed under the electron microscope, and designated ϕRs551 (Fig 1). This bacteriophage has an average size of ~1200 nm in length and ~7 nm in width, similar in morphology to other bacteriophages of *R*. *solanacearum* isolated from soil, such as ϕRSM1 (1500±300 nm in length, 6±0.7 nm in width) [8] and ϕRS603 (1120 nm in length, 8 nm in width) [9].

To determine the host specificity of ϕRs551, 12 *R*. *solanacearum* strains including nine r3b2 and three non-r3b2 strains originally isolated from different parts of the world were tested for their susceptibility to the phage (Table 1). ϕRs551 infected two of the nine r3b2 strains, RUN628 and 4155, and one of the three non-r3b2 strains, RUN302 (Table 1), as evidenced by the formation of plaques on CPG plates layered first with the tested *R*. *solanacearum* strains and spotted later with ϕRs551.

### Genome characterization of ϕRs551

**Fig 2:**
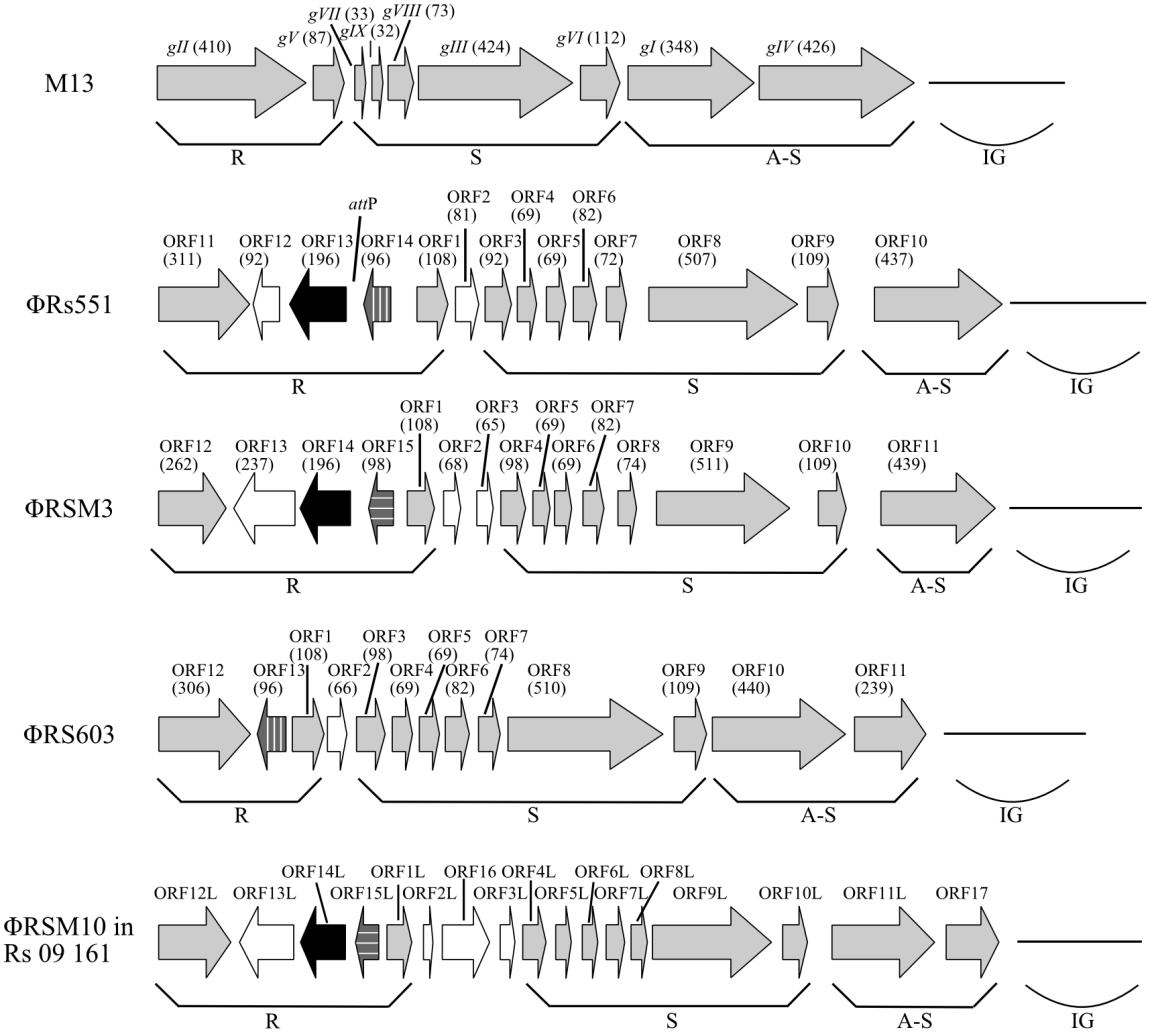
Genomic organization of the phage ϕRs551 and the prophage RSM10, with reference to M13 for general structure and comparison to related *Ralstonia* phages ϕRSM3 and ϕRS603. The numbers in parentheses indicate the length (aa) of the ORF. The arrows represent the direction of transcription of ORFs. ORFs similar to the ones in M13 are in gray. ORFs annotated as a putative integrase and phage repressor are in black and darker gray, respectively. Types 1 and 2 putative phage repressors are differentiated with horizontal lines for the former and vertical ones for the latter within the darker grey arrows. ORFL stands for ORF-like in ϕRSM10. The location of the core *att*P site is indicated only in ϕRs551.

The genome of ϕRs551 was degraded by DNase I, but not by RNase A. It was also degraded by S1 nuclease, specific for circular single stranded nucleic acids, but not by Exonuclease I, which cleaves nucleotides from the end of a polynucleotide chains. The complete nucleotide sequence of ϕRs551 was determined (S1 Fig) and submitted to GenBank (accession no. KX179905). The genome consists of 7929 nucleotides and has a G+C content of 61%. Fourteen putative open reading frames (ORFs) were identified in ϕRs551 and compared by BLASTP to genome sequences of other *R*. *solanacearum* filamentous phages including ϕRSM1, ϕRSM3, and ϕRS603 (Table 3). Each of the ORFs is capable of encoding a protein with a molecular mass of greater than 7.5 kDa. The deduced ORFs, their positions, putative functions, and identities to the closest *R*. *solanacearum* phage homologues are summarized in Table 3. The predicted ORFs could be arranged in a similar modular structure to that of previously characterized *R*. *solanacearum* filamentous phages of the Ff group ϕRSM3 and ϕRS603 [9,20], with structural differences in two variable regions; one region located between the replication module and the structural module and the second within the replication module (Fig 2). Between the replication module and structural module, ϕRs551 has only one predicted ORF, similar to ϕRS603, but different from ϕRSM3 which has two ORFs (Fig 2). Within the replication module, ϕRs551 has three reverse-oriented ORFs, 12, 13 and 14, as in ϕRSM3 (ORFs 13, 14 and 15). This is different from ϕRS603, which only has one reversed ORF, ORF13, corresponding to the ORF14 of ϕRs551 and the ORF 15 of ϕRSM3. ORF13 of ϕRs551 was annotated as a putative resolvase (ORF14 in ϕRSM3) that ϕRS603 does not have. ORF14 in ϕRs551 was annotated as a putative phage repressor, similar to the annotation for the ORF13 in ϕRS603 and the ORF15 in ϕRSM3, but the ORF14 in ϕRs551 shared 81% homology to ORF13 in ϕRS603, and no significant homology to ORF15 in ϕRSM3.

**Table 3:** List of annotated ORFs of ϕRs551and their BLASTp results.

*Coding sequence*	*Strand*	*Position 5’-3’*	*Length of Protein (aa)*	*Amino acid sequence identity/similarity to best homologs (Query cover %; no*. *of amino acid identical; % identity)*	*E-value*	*Accession no*.
ORF1	+	12–338	108	Hypothetical protein–*Ralstonia solanacearum* CMR15 (99; 104; 96)	3e-70	CBJ37262
ORF2	+	338–583	81	Hypothetical protein ORF2– RS603 phage (72; 48; 72)	7e-17	BAP74427
ORF3	+	593–871	92	Hypothetical protein ORF4– RSM1 phage (100; 56; 57)	3e-18	BAF36510
ORF4	+	871–1080	69	Hypothetical protein ORF4– RS603 phage (70; 41; 84)	5e-19	BAP74429
ORF5	+	1080–1289	69	Hypothetical protein ORF5– RS603 phage (100; 66; 96)	2e-37	BAP74430
ORF6	+	1292–1540	82	Hypothetical protein ORF7– RSM3 phage (98; 80; 98)	6e-53	BAG75139
ORF7	+	1543–1761	72	Hypothetical protein ORF8– RSM1 phage (98; 69; 93)	3e-14	BAF36514
ORF8	+	1836–3359	507	Hypothetical protein ORF9 –RSM3 phage (99; 414; 81)	0.0	BAG75141
ORF9	+	3372–3701	109	Hypothetical protein ORF9– RS603 Phage (99; 102; 94)	6e-57	BAP74434
ORF10	+	3706–5019	437	Zonular occludens toxin ORF11 –RSM1 phage (99; 393; 90)	0.0	BAF36517
ORF11	+	5537–6472	311	Rolling circle DNA replication initiation protein–*Ralstonia* phage 1 NP-2014 (99; 281; 90)	0.0	AHI87735
ORF12	-	6469–6747	92	Hypothetical protein–*Ralstonia solanacearum* CMR15 (98; 67; 73)	2e-32	CBJ37251
ORF13	-	6767–7357	196	Resolvase–*Ralstonia* phage 1 NP-2014 (99; 167; 85)	2e-118	AHI87737
ORF14	-	7458–7748	96	Lambda repressor-like—ORF13 RS603 phage (97; 77; 81)	1e-49	BAP74438

### Presence of ϕRs551 genome in *R*. *solanacearum* strains and Phylogenetic analysis with well-identified *R*. *solanacearum* filamentous phages

The ϕRs551 nucleotide (nt) sequence was found with 100% identity in 11 deposited genomes of *R*. *solanacearum* strains: UW551, UY031, IPO1609, NCPPB909, CFIA906, UW24, UW365, RS2, Geo_56, Geo_96, and Geo_99. The first eight strains were deposited as r3b2 strains, although the race and biovar nature of the last three unpublished strains was unknown. Such phage sequence, however, was not found in two other *R*. *solanacearum* strains deposited as r3b2: NCPPB282 and 23-10BR. Askora et al. [15] previously identified a prophage sequence in UW551, putatively naming it ϕRSM4, and similarly predicated the ORFs [15, 20], but no research was conducted beyond its annotation. We named our ORFs in ϕRs551 consecutively from ORF1 to 14 and annotated ORF2, ORF3 (ORF4 in ϕRSM4), and ORF13 (ORF14 in RSM4) to have 81, 92, and 196 instead of 59, 98, and 237 amino acids, respectively. ϕRs551 is most closely related to the prophage RSM6 identified in the phylotype III *R*. *solanacearum* strain CMR15 [20] and the phage ϕRS603 [9], matching 89% and 83% of the genome with 88% and 82% identity at the nt level, respectively. ϕRs551 differs from the prophage RSM6 in the middle of ORF 8, which could have an impact on host range [15], as well as in ORFs 2, 3, and part of the intergenic region. The difference between ϕRs551 and ϕRS603 is that ϕRs551 has an annotated resolvase that is not found in ϕRS603 (ORF13), but is missing a second ORF in the A-S module (Fig 2) that ϕRS603 has in common with ϕRSS phages [9].

Interestingly, the nt sequence of ϕRs551 also matched genome RS-09-161, a *R*. *solanacearum* strain isolated from India [34], with 84% identity to 74% coverage of ϕRs551. Inspection of this homologous RS-09-161 sequence revealed a previously unidentified prophage of 9947 nt (position 3189 to 13135 in GenBank accession number JHBO01000069) with 17 predicted ORFs, which is two to four more ORFs than the other *R*. *solanacearum* Ff-type phages including ϕRSM, ϕRs551 and RS603 (Fig 2). We designated the newly identified prophage ϕRSM10 to continue the naming and numbering system used by Yamada’s group for prophages identified previously in *Ralstonia* species [20]. The ϕRSM10 sequence has an insertion in the replication module that is similar in structure to ϕRSM3 and ϕRs551 (Fig 2). The structure of its A-S region is similar to ϕRS603 and ϕRSS phages [9]. Also, there is an additional ORF (ORF 16) (position 4188 to 4808 in JHBO01000069) (Fig 2) between the replication module and structural module that is not found in any other ϕRSM or ϕRSS phage and has 85% identity to a hypothetical protein in *Methylomicrobium album* BG8 (Accession number EIC30310). ϕRSM10 also differs from ϕRs551 in the repressor region, where ORF15L in ϕRSM10 (position 3240 to 3554 in JHBO01000069) is 100% identical to ORF15 in ϕRSM3 (Fig 2).

### Integration mechanism of ϕRs551

**Fig 3:**
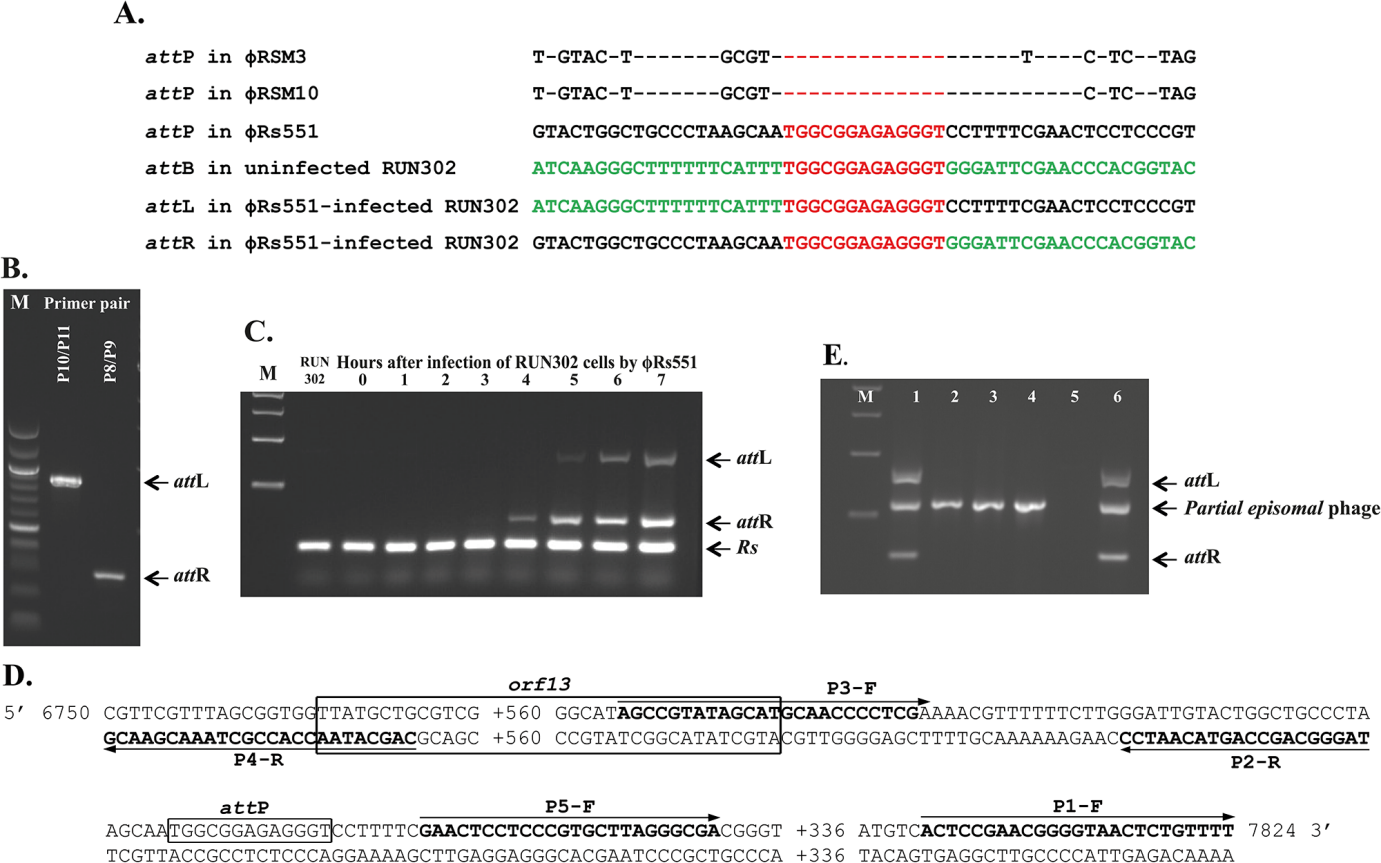
Integration of ϕRs551 into the genome of *R*. *solanacearum* strain RUN302. (A) Comparison of the attachment (*att*) sequences. The *att*P sequences identified in ϕRs551 and the prophage RSM10 are compared with that in ϕRSM3 of *R*. *solanacearum*. The 13-nucleotide core *att* sequence is shown in red. For prophage RSM10 and ϕRSM3, only nucleotides different from the ones in ϕRs551 are shown. The *att* sequences before (*att*B) and after ϕRs551 integration into *R*. *solanacearum* strain RUN302 (*att*L and *att*R) were shown. Sequences from the bacterial strain RUN302 and the phage ϕRs551 are shown in green and black, respectively. (B) After integration, the presence of the left (*att*L) and right (*att*R) integration flanking regions in ϕRs551-infected RUN302 were amplified by PCR with primer pairs P8/P9 and P6/P7, respectively, and separated by gel electrophoresis. (C) The *att*L and *att*R regions were amplified by PCR every hour for seven hours after infection of *R*. *solanacearum* RUN302 with ϕRs551 to determine the time needed for phage integration to occur. Note that no *att*L and *att*R fragments were detected in the uninfected wild type RUN302 strain, although the *R*. *solanacearum* (Rs) fragment was detected in all strains/time points by PCR with primer pair P10/P11. (D) PCR amplification generated ϕRs551 mutants consisting of shortened phage regions using primer pairs: P1-F/P2-R to obtain ϕΔ*att*P missing a 395-bp fragment containing the core *att*P sequence, P3-F/P4-R to obtain ϕΔ*orf13* missing 570 of the 591 nucleotides of the *orf13* gene sequence, and P5-F/P4-R to obtain Δ*att*P-Δ*orf13* missing a 655-bp region containing both the core *att*P and 582 nucleotides of the *orf13* gene sequences. The primer sequences are in bold, and their directions are indicated by arrows. The *orf13* gene and the 13-nucleotide core *att*P sequences are boxed. Five hundred and sixty nucleotides between P4-R and P3-F, and 336 between P5-F and P1-F are not displayed. (E) Deletion of ϕRs551’s *att*P or/and ORF13 (the integrase) abolished its ability to integrate in the genome of *R*. *solanacearum* strain RUN302. Lanes: RUN302 infected by the wild type ϕRs551 (1), the single mutant phage ϕΔ*att*P (2), ϕΔORF13 (3), the double mutant phage ϕΔ*att*P&ΔORF3 (4), and the full-length phage ϕRs551 generated by PCR (6), as well as uninfected wild type RUN302 (5). M, 1 kb DNA ladder. Arrows indicate the fragments for *att*L, *att*R, Rs and the partial episomal phages.

The discovery that the full sequence of ϕRs551 was present in 11 deposited genomes of *R*. *solanacearum* strains in GenBank suggests that ϕRs551 has a lysogenic ability and is able to integrate into the host genome. By BLAST search, we identified a possible *dif*-like sequence for *att*P in ϕRs551 that contains a 13-bp core sequence, 5′ TGGCGGAGAGGGT 3′, corresponding to nucleotides 7410 to 7422 in ϕRs551, as well as a putative resolvase encoded by ORF13. This core sequence showed 100% identity to the *att*P sequences of other *Ralstonia* RSM phages including ϕRSM3 and our newly identified prophage ϕRSM10 (Fig 3A), as well as the *att*B sequence of *R*. *solanacearum* strains such as RUN302 (Fig 3A). To confirm that this core sequence is involved in the integration of ϕRs551 into its susceptible host strain RUN302, we amplified and determined the left and right integration flanking regions, *att*L and *att*R, by PCR in ϕRs551-infected Run302 (Fig 3A and 3B). Both the *att*L and *att*R fragments contained the 13-nt core *att* sequence that is in both the bacterial RUN302 and the viral ϕRs551 genomes (Fig 3A). The *att*L site had RUN302 genome sequence upstream and ϕRs551 sequence downstream of this core sequence. Reversely, the *att*R fragment contained ϕRs551 sequence upstream and Run302 sequence downstream of the core sequence, suggesting that the core sequence is where the phage integration happened.

The time needed for ϕRs551 to integrate into the RUN302 genome was determined to be between four to five hours after phage infection, since the integration flanking regions *att*L and *att*R were not amplified until five hours after RUN302 was infected by ϕRs551 (Fig 3C). A 147-bp bacterial genome target, however, was detected in the wt and ϕRs551-infected RUN302 genome regardless of phage integration (Fig 3C).

To determine the role of ORF13 and to further investigate the importance of the core *att* sequence (*att*P) in the integration of ϕRs551 into its susceptible *R*. *solanacearum* host strain RUN302, two single phage mutants, ϕΔ*att*P missing the *att*P and ϕΔ*orf13* missing the ORF13 gene, and one double mutant, ϕΔ*orf13*&Δϕ*att*P missing both the *att*P and the ORF13 gene, were generated (Fig 3D). None of the phage mutants integrated into the genome of RUN302 after infection, since no *att*L and *att*R fragments were detected by PCR using primer pairs P10/P11 and P8/P9 (Fig 3E). Instead, they replicated as circular episomal forms, since they were amplified by the primer pair P14/P15 that target ϕRs551 regions away from the mutated regions, and yield PCR products only if the phages were circular and outside of the bacterial genome (Fig 3E). Both the wild type ϕRs551 and the ϕRs551 generated by PCR existed as both lysogenic and episomal forms, as evidenced by amplification of *att*L, *att*R and the partial episomal ϕRs551 by primer pairs P10/P11, P8/P9 and P14/P15 (Fig 3E).

### Physiological effects of ϕRs551 on *R*. *solanacearum* strain RUN302

**Fig 4:**
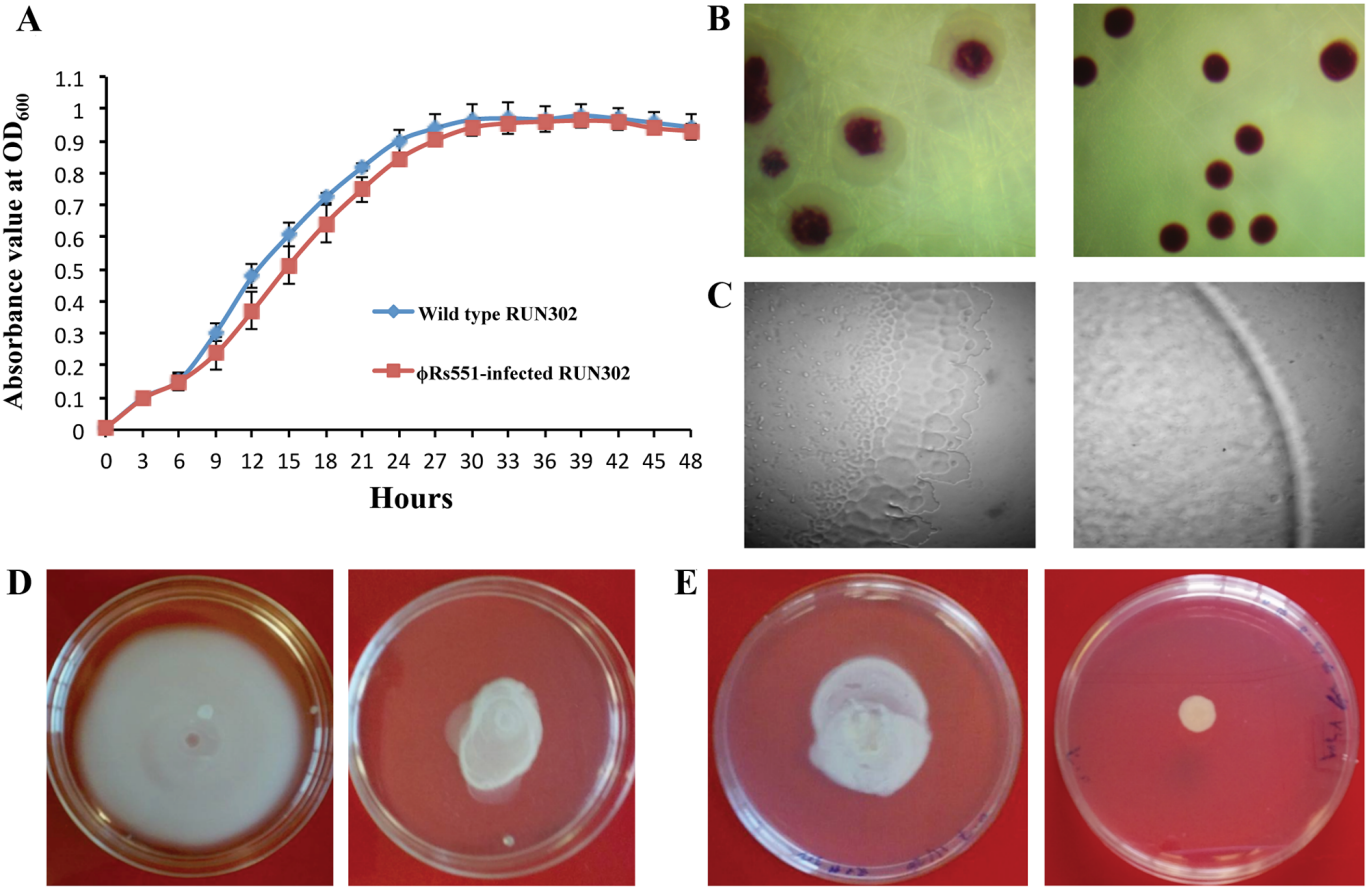
Impact of ϕRs551 infection on *R*. *solanacearum* strain RUN302 host cells. Growth in CPG liquid medium (A) and on TZC plates (B), twitching (C), swimming (D), and swarming (E) activities of the wt (diamonds in A, left in B to E) and ϕRs551-infected (squares in A, right in B to E) RUN302 strains. Bacterial growth in CPG was measured at the absorbance of 600 nm and graphed every three hours for 48 hours. Bars indicate standard errors. Three microliters of bacterial suspension (10^8^ cells/ml) were inoculated in the center of the twitching (minimal agar medium), swimming (CPG with 0.3% (w/v) agar), and swarming (CPG with 0.7% (w/v)) assay plates, incubated in the dark at 28°C and photographed five days after inoculation (D and E) under a microscope (C).

To determine the effects of ϕRs551 infection on *R*. *solanacearum* strain RUN302, we first compared the in vitro growth of the wild type (wt) with that of the ϕRs551-infected strain RUN302. The growth curve of the two strains was measured every hour for 48 hours, and graphed every three hours (Fig 4A). Although the wt RUN302 grew faster during the exponential growth period (9 to 27 hours) than the phage-infected strain, the difference was not significant at all but one (12 h) time point. Overall, the two strains grew at a similar rate during the entire 48 hour period (Fig 4A). When the two strains grew on regular TZC medium plates, the colonies of the wt strain grew irregular and fluidal, while the phage-infected strain appeared circular and dry (Fig 4B), suggesting low production of EPS. This was confirmed by an EPS quantitative assay that showed ϕRs551-infected cells produced significantly lower amounts of EPS (71.5±3 mg/10 ml) than the wt cells (107±5 mg/10 ml). The two strains also displayed distinct difference in motilities when compared using the twitching, swimming and swarming motility assays (Fig 4C, 4D and 4E). For the wt strain, under a microscope, we observed the formation of corrugated trajectories around the edge of its colonies, indicating twitching motility (Fig 4C, left). Such trajectories, however, were largely missing in the phage-infected RUN302 strain and the edge of its colonies was smooth (Fig 4C, right). Our assay for swimming activity revealed that the wt RUN302 expanded almost to the entire plate five days after inoculation of 3 μl of the bacterium in the center of the plate (Fig 4D, left), while the phage-infected RUN302 strain did not expand to even half of the plate radius (Fig 4D, right). Similarly, in our swarming assay, the wt RUN302 strain was observed to swarm over half of the plate radius (Fig 4E, left), while the phage-infected strain only moved approximately one third of the distance as compared to its wt (Fig 4E, right).

### Effects of ϕRs551 on the virulence of *R*. *solanacearum* strain RUN302

**Fig 5:**
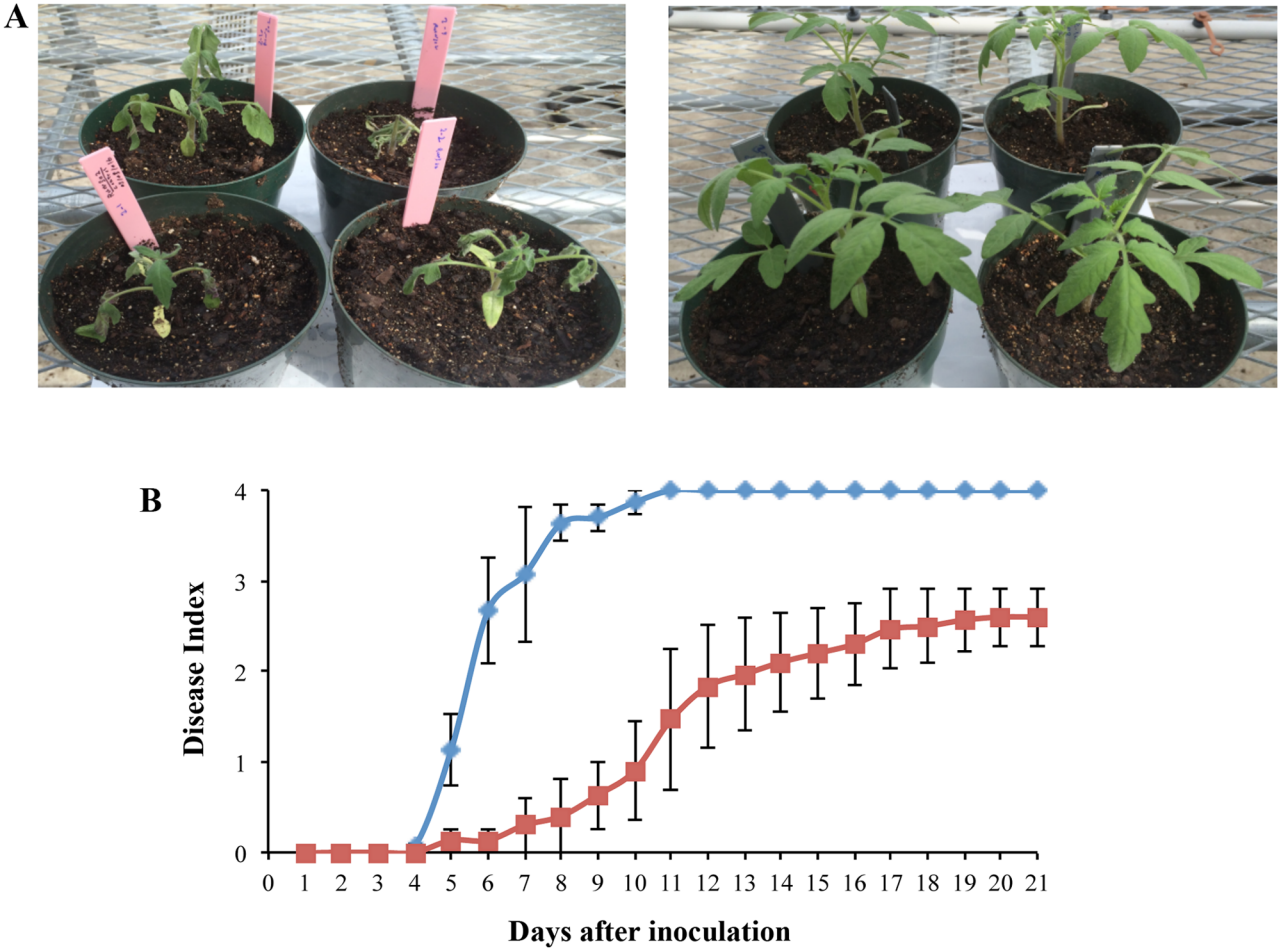
Effect of ϕRs551 on virulence of *R*. *solancearum* strain RUN302 in tomato. Three-week-old tomato plants were inoculated by pouring 50 ml (5 x 10^7^ cells/ml) of wild-type or ϕRs551-infected *R*. *solanacearum* RUN302 strain into each pot. A, appearance of the inoculated plants 10 days after inoculation with the wide type (left) or ϕRs551-infected (right) RUN302 strain. B, virulence of the uninfected wild type (diamonds) and ϕRs551-infected (squares) RUN302 strains. Plants were rated on a disease index ranging from 0 (healthy) to 4 (76 to 100% leaves wilted) [31]. Points shown are means of three separate experiments, each containing 10 plants per treatment. Bars indicate standard errors.

To study the effect of ϕRs551 on the virulence of its susceptible strain RUN302 of *R*. *solanacearum*, we compared the virulence of the wild type to that of the ϕRs551-infected RUN302 of *R*. *solanacearum*. The wild type strain RUN302 started to cause disease symptoms four days after soil drenching inoculation (disease index (DI) > 0), reached a DI of 2 (50% of leaf area wilted) in fewer than six days, and completely wilted all inoculated plants (DI = 4) by day 11 (Fig 5). On the contrary, the virulence level caused by ϕRs551-infected RUN302 strain of *R*. *solanacearum* was significantly lower (Fig 5). It did not cause any disease symptoms until five days after inoculation, took more than 14 days to cause a DI of 2, and was unable to cause any disease symptoms on some inoculated plants even 21 days after soil drenching inoculation (Fig 5).

## Discussion

We purified an Ff-like filamentous phage ϕRs551 from the supernatant of *R*. *solanacearum* strain UW551 grown under normal growth conditions. We also characterized ϕRs551’s morphology and genome, and determined its integration mechanism and effect on the morphology, EPS production, in vitro growth, motility and virulence of its host bacterial cells. Different from previously isolated filamentous *R*. *solanacearum* phages, ϕRs551 is the first active virion identified and purified directly from the supernatant of a normally grown *R*. *solanacearum* strain without any form of induction [35], rather than from the soil of a crop field like ϕRSM1 and ϕRSS1 [8] or regenerated like ϕRSM3 from a prophage in a lysogenic *R*. *solanacearum* strain [15]. In addition, the genome sequence of ϕRs551 was found with 100% identity to its prophage sequence in the deposited genome of *R*. *solanacearum* strain UW551 from which the phage was purified. Surprisingly, when the genome sequence of ϕRs551 was used as a query sequence to blast GenBank’s nucleotide collection, NCBI genomes and whole genome shotgun contigs databases, the sequence was also found with 100% identity in the genomes of ten other *R*. *solanacearum* strains sequenced and deposited independently by different research groups: Geo_57, Geo_96, Geo_99, UY031, IPO1609, NCPPB909, CFIA906, UW24, UW365, and RS2. The race and biovar nature of strains Geo_57, _96 and _99 were not given in GenBank, but the other eight strains including UW551 are all deposited as r3b2 strains. All 11 strains, however, belong to phylotype IIB, sequevar 1 as confirmed by Stulberg et al. [32] for UW551 and IPO1609, reported by Clarke et al. [36] for NCPPB909, POPS2 (alternative ID of UW365) and RS2, and determined in this study for Geo_57, Geo_96, Geo_99, UY031, CFIA906 and UW24 using a *R*. *solanacearum* typing computer program [37]. Such phage sequence, however, was not found in two other genomes deposited as r3b2 strains: NCPPB282 and 23-10BR, although the former belongs to phylotype IIB sequevar 2 [36] and the latter with an alternate ID of UW349 was determined previously by Stulberg et al. [32] to be an atypical biovar 2 strain, belong to phylotype IIB sequevar 27 and not as cold virulent as UW551. The ϕRs551-containing r3b2 strains were originally isolated from different countries at different times—Kenya/US in 2003 (UW551), Uruguay in 2003 (UY031), the Netherlands in 1995 (IPO1609), Egypt in 1961 (NCPPB909), an unknown source and time (CFIA906), Israel in 1955 (UW24), China in 1980 (UW365) and Bolivia in 2008 (Rs2) [36], suggesting that the phage infection might have occurred in a common ancestor of the *R*. *solanacearum* strains and been stably maintained over the years after the strains were disseminated in Asia, Africa, Europe and South America. It is also possible that ϕRs551 released from the ancestral lysogenic *R*. *solanacearum* strain infected local susceptible *R*. *solanacearum* strains when the ancestral strain was disseminated into different geographic locations. This is different from other *R*. *solanacearum* filamentous phages, where their prophage sequences in *R*. *solanacearum* were similar or closely related to the phages, but not exactly the same. For example, the prophage ϕRSM3’s sequence was identified in *R*. *solanacearum* strain MAFF730139, and is 75 bp shorter than and had 93% nucleotide identity to the isolated phage ϕRSM1 [15]. Currently, a leading hypothesis of the origins of r3b2 strains posits that they disseminated out of South America and are largely clonal [36, 38]. Since ϕRs551 has been identified in the genomes of sequevar 1 strains, its presence or absence might be useful for tracing the roots of the r3b2 strains of *R*. *solanacearum*.

Askora et al. [16] provided the first evidence that the ORF14 of the *R*. *solanacearum* filamentous phage ϕRSM1 functioned as a small site-specific serine recombinase from the resolvase/invertase subfamily for integrative and excisive recombination of the phage in *R*. *solanacearum*. They used an indirect approach for the study by cloning ϕRSM1’s ORF14 and *attP* sequences into an *E*. *coli* plasmid and observed integration of the resulting plasmid pT-orf14-attP into the *attB* sequence of *R*. solanacearum [16]. They also observed the occurrence of an intermolecular recombination between the *E*. *coli* plasmid pT-orf14-attP and a *R*. *solanacearum* plasmid containing *attB* in *R*. *solanacearum* cells [16]. In our study, we used a direct approach by constructing ϕRs551 mutants missing orf13 and *attP* either alone or in combination, and found that all three phage mutants ϕΔorf13, ϕΔattP and ϕΔattP&Δorf3 were unable to integrate into RUN302 (Fig 3E). Our study, therefore, confirms that, similar to ϕRSM phages, ϕRs551 relies on its own ORF13 (ORF14 in ϕRSM1 and ϕRSM3), a resolvase, and a core *att* sequence for site-specific integration into its susceptible *R*. *solanacearum* host genome. It is worth mentioning that the filamentous *R*. *solanacearum* phage ϕRS603 has not been found in an integrated state [9], and a counterpart resolvase of the ORF13 of ϕRs551 or ORF14 of the ϕRSM has not been identified in ϕRS603, further suggesting the involvement of a resolvase in the integration of *R*. *solanacearum* filamentous phages.

ϕRs551 maintained both prophage and free particle states, unlike *R*. *solanacearum* RSM and RSS phages in which episomal DNA and virions were rarely identified once a prophage state was established in the lysogenic strains [39]. The phage ϕRs551 appeared spontaneously in the supernatant of the *R*. *solanacearum* lysogenic cells and was able to replicate to a rate of 10^11^pfu/ml even after the prophage state was established in RUN302. Yamada [39] hypothesized that the prophage state and phage immunity in the RSS and RSM phages were possibly maintained by the regulatory function of the putative phage repressor encoded by the orf13 of ϕRSS and the orf15 of ϕRSM. Interestingly, the putative repressor gene identified in ϕRs551 and encoded by the ORF14 showed no homology to the putative repressor of ϕRSM3 which we named a type 1 repressor, but shared a high homology (81%) with the putative repressor of ϕRS603 which we named a type 2 repressor, suggesting that the type 2 repressor may not suppress phage replication as strongly as the type 1 repressor. Our naming of the two types of putative repressor proteins is consistent with previous findings that ORFs encoding for the putative repressors in phages ϕRSM1 and ϕRSM3 and the prophage RSM7 shared weak homology with the ones in the prophages RSM4, RSM5 and RSM6, as well as in the phage ϕRS603, although the ORFs within each of the two groups shared high amino acid similarity [9,20]. It is worth mentioning that ϕRs551 is the first isolated virion that contains an integrase (ORF13) and a putative type-2 phage repressor (ORF14), since the RSS and RSM phages contain an integrase but a type 1 repressor and the RS603 phage contained a type 2 repressor but did not encode any integrase. The significance of such an integrase and repressor combination is unknown, and the biological functions and competiveness advantages, if any, such a combination offers to the phage and its host bacterium will be the focus of further study.

Infection of a *R*. *solanacearum* host strain RUN302 by ϕRs551 resulted in less fluidal bacterial colonies with significantly reduced production of the extracellular polysaccharide, a major virulence factor [40]. The overall in vitro growth of the bacterium in liquid CPG, however, was not significantly affected. This is similar to RSM3-infected *R*. *solanacearum* cells that had similar growth curve to their uninfected wild type cells [19]. The phage infection also reduced the bacterium’s twitching, swimming and swarming motilities, which are important virulence factors, especially during root invasion and early colonization [41,42]. As a result, at least partially, it is not surprising that the virulence of the ϕRs551-infected *R*. *solanacearum* was significantly reduced, although the reduced virulence is different from the loss of virulence caused by infection with the filamentous RSM-type phage ϕRSM3 [19]. Addy et al. [19] demonstrated that the virulence can be restored when the putative phage repressor encoded by the ORF15 of ϕRSM3 was deleted, suggesting negative regulation of virulence by the ϕRSM3-encoded repressor protein. Since the putative phage repressor in ϕRs551 (a type 2) is different from the one in the RSM phages (a type 1), the putative repressor in ϕRs551 may not regulate virulence as tightly as the one in ϕRSM3, resulting in a significantly reduced but not loss of virulence in ϕRs551-infected *R*. *solanacearum* cells in tomato plants. Our host specificity study revealed that ϕRs551 infected a few but not all tested r3b2 strains, as well as a closely related non-r3b2 sequevar 4 strain of *R*. *solanacearum* (Table 1), suggesting that ϕRs551 is a more selective phage such as ϕRSM1 [15]. This combined with the reduced, not complete loss of virulence caused by ϕRs551 argues against its potential as a biocontrol agent by itself. Future research, however, is needed to study the roles, if any, played by the phage in other aspects of its host bacterium. For example, will producing and releasing ϕRs551 virions offer any fitness or competitive advantage to the *R*. *solanacearum* lysogenic strains by infecting susceptible *R*. *solanacearum* strains occupying the same environment, competing for the same ecological niche for plant infection and survival? Better understanding of the phage-bacterium-environment interactions will facilitate the development of integrated management strategies to combat *R*. *solanacearum*. Future research is also needed to determine the regulatory role played by the putative repressor of ϕRs551 in integration and excision of the phage, as well as in virulence and competiveness fitness of its bacterial hosts.

In this study, a new prophage RSM10 was identified from the deposited genome sequence of *R*. *solanacearum* RS-09-161. Although its entire sequence is homologous to a combination of ϕRS603, ϕRs551and the RSM phages, its structure in the A-S region is similar to ϕRS603 and ϕRSS phages, suggesting that the RSM10 prophage might be an evolutionary intermediate between ϕRSM and ϕRSS phages. The discovery that the RSM10 prophage also contains an ORF that is homologous to a hypothetical protein in *Methylomicrobium album* suggests that genetic material has been exchanged; although it is unknown how this horizontal gene transfer has occurred. It is worth mentioning that the putative repressor (ORF15L, Fig 2) annotated for the new prophage RSM10 is 100% identical at the amino acid level to the putative repressor protein (ORF15) of the prophage RSM3 (Fig 2), suggesting that different from ϕRs551, ϕRSM10 contains a type 1 repressor (Fig 2). Whether an active virion of RSM10 can be isolated from *R*. *solanacearum* strain RS-09-161, what roles played by the type 1 repressor in ϕRSM10, and what are the effects of the prophage on the evolution and host virulence in *R*. *solanacearum* remain to be determined.

## Supporting information

S1 Fig Complete nucleotide sequence of ϕRs551. (DOCX)
