# Supplementary material for: Molecular and biological characterization of ϕRs551, a filamentous bacteriophage isolated from a race 3 biovar 2 strain of Ralstonia solanacearum
Source: PLoS One. 2017 Sep 21;12(9):e0185034. doi: 10.1371/journal.pone.0185034 (PMC5608472; doi:10.1371/journal.pone.0185034)
Supplement: S1 Fig — (DOCX) [file pone.0185034.s001.docx]

1 TCGGAGCCAT CATGTCGAAC ACTCAAAAGC TGACCATCAT CGCCATCAAC AGCCGTAATG GCGTGTCCGC AAAGACGGGC

81 CGCCCGTACT CGATGCACGA AGCGCAATGC ATCCTCACCG AAGGTGTTGC TGACGCAACT GGCGGCGTCT CCGAACAGAT

161 CAAGGTTGGC CGCGTGAACG TCGCCGACGA ACTCAAGGAC ACCGTACCCG GCGACTACGT GGCCGACTTC AAGCTGTTCG

241 TCTCGCGCGA CGGAGAGCTG GTCGCGCGCA TCGTTGGTCT CAAGGCGCTG ACGGTCTCTC GCCCCGTCCC CTCCTCCGCC

321 GAAAAGAAAG CGGCCTGATG CTGATCCATG CGAAGCCCGG CGATGCCGGG CTTTTCCCGT TAACGGGCGT TCAGGAGGTC

401 GTCATGGTCG ATCCGAACGA CAAGTACACG ATGCAGTTGC CGCTGATGCG TTATCGCGCG GGGCTGCGTC TGCGGGAGTT

481 TCTGGACTAT CGCGACTGGG CGGGTTGTGA GGTCTCGTTC CAGTGCAACG AGTTCGCCGC GGCGGCGTTG CAGGCGATGG

561 GCGGCATGCT GGGTCGTTGG TAGGAGGTCG TGATGGGCTC GTGCGTCAAT GCTTTGGTTA TGGCGCTGTT CGTTCTGCTG

641 CTGACGCTGT TGGTCCCGGC GTGGGCTGTC TGGAAGAGTT CAGGGGCGTT CTGGTCGGGG GCGTCGTCTG CATGGCTCGG

721 CTATCTGTGT CGGGAGCGTG GCGAGCTGCT GACGGCGTTG GCTCTTCGCG ATGAGGCGTA CAGCGCCTTG GACGGCAAGG

801 GGCTGGAGGT TGCTGATGTT CTGGCACAGC TTGCCCTGGA ACGCCTCGGT GGTCTTGCGG GGGAATGGTG ATGAGGCTGC

881 CTAGCTGGCT CTGGTTGCTG CTGGTCGGCT GGCTTGCGAC AGCAGTTGCG CATCACGCGT GGCGCTTGTT CTCAGTGATC

961 GGTTTCTCTG GCGTGTCGAT GTTGATTCAC GGCGGGCATG CGGTGGCTAG AGCGTGTTTG CTGCTGGTGG TGGGCGTGAT

1041 GGTCGGCCGT CTGGTGCGGC GTCTTTTTCG GAGGTCGTGA TGTCAACCCT TGCTGAGATC GAAGCTCAGA TCGCTGAGCT

1121 GGAAGAGGCC AACGAGAACT TGCACGACTC GTTGGATGCC GCTGAGGCGA TGGACGACGC TGAGGCTATC GAGTTCTTCG

1201 GCAGCCTGGA TGAGCGTGAT GAGGCGATTG AGGCCTGGGA GGCGGCGATC CAGTCGAACC TGGAGCAGAT TGCCGAGCTG

1281 GAAGGCTGAT CGTGGCTCAG TGCGTAGTGA TCCAAAACGG TGGGTTGGTC TTCACGACCG ATCCGCCACA GAGCTGCAGC

1361 GGCTATCTGC TGTTGCAGCC GAGCGAATAC TCGAACGTGA TGGCTCTGTC CGGCGCGTTC ACGTATCCCA GCGCGAGCGA

1441 TTTTGCGGCC GCGTTTACCG CTGGGTTTCA ATGGCCGGTG TTCTTCTTCA TCGTTGCAAT GCTGGTTTCC AAGGTCGCTT

1521 CCTTCTTCGA TAGGGGTTGA ACATGAAACT GACTCAAAAA CTGGCCGCTG GTGCGGCTCT GGCCGTGGGC GGCGCGGGTG

1601 CTGCGATGGC GCAAACCACG ACCAGCGGTA TCGATTTCTC GTCCATGACG GGTGCCGTGA GCGCGACCGC TGTGGTGGCT

1681 GCGCTGGTGG CCATGGGCGT GGTCAAGATC GGTCCGGGCT TCGCCAAGTG GGCGCTGAAC AAGGTTGCGG CGTTCTTCTG

1761 ATCGGCGGCG TTGACGTAGT ACGGCCCAGT TGCTCCCCGG CGCTGGGCCT TTTTCGTTTC TGGAGGTCAC ATGCTATGGC

1841 TACTGTTCTG GGGTTGGATC GGCTCGCTGT GCGTGCTGGC GGTCATGCTC GGCTGGGAAA TTGGCTCGCG CTGATCGTCG

1921 CGTGCGTGTT GTCTTTAGGG ACCGCGCAGG CGCAGTCGAA CGGTTTGGAT TTGACGGTCA AGCAGAGTGG TCCCTCTGTT

2001 CCGCAGACGC CATCCGGCAA TGTTATGTCG GTTCTGGTGC CAATCACCAT AGGTGTCGTG GCGGTCGGCG CTGCTGCGGT

2081 GGCGTTGCCG GCCACTGGCG CGCTGGCCAT CACGGGCGAC GTGATTGCTG CGGCCGGCTC GTCGGCGATC CGTAAGGGCG

2161 TCATCAATGG AGCGGCGCTA GCGGGCATCG TGGCGCTGAT CGGGGCACCG TCGGGTATTT CGCTCGATTC AGGCGGGAGC

2241 ATGGTGGCGC CTGCGGTGTC GGCCAATGCC GGCGACGCTG GTTTCAACGG GTTCGGCTGG GGGTATTCGT ACAACACGTC

2321 TGTGTCGGGC GGCAGTTACG GTAATGGCGT CGCTGCGTCT CCTGGGGCGG CCTGCGCTGC GATGTTGGCG GCGGACGCGT

2401 ATCTGGCGTC GAATAACGCC AAGCTAGCGG GTATTCGGCC GACGGGGACG TCTTACGAAT GCCATTTCAC TAACGATGGC

2481 GGTTCGAACT TCTATTCCGG TGTTGGTCCG ACTGGCAGCT GCATCAGCGG CTACGTTGCG TCCGGAAGTT CTTGCGTGCC

2561 GGATCAGTCT GGGCCGAAGC AGGCTGCGAC TGACGCGCAA ATTCAGTCGG CGATCAAGGC AGCGCCGGCT AGCTGGCCAT

2641 CGGTCTACAA CAATGCGGGC TGCCCTTCCG TCAACACGAT GACGAACGTT GTAGGCAGCG GTTCGAACGA TCCGTGCGCA

2721 CAGATGATTG GGGCTCCGTC GACGGGGTAC GGGGTTTCGT TTCCGTCGGG CAACACCGTC GCCGGTACGC CGAAGACCGA

2801 CACGCAGACG AAGGTGAATG CGGACGGCAC GAAGACGACG ACGAACACGA CAACCAACAC GACGACGACG CTCACGGGTA

2881 CACAGGATCG CGTGAACCCG GTGGAGGGCA CGACGACGAC GAGCACGTCG GTGTCGACCA CGACGACGAA CCCTGATGGC

2961 AGTACGACGA CGACTACTAC CACGACCACG GATCAGGCTC CGCCGGCGAC GGCTTCGAAC CCTGCCAACG AGCAGCAGCA

3041 GTCGACGACT GCGACGTTTG CGGCTCCGGA TACTTCGCTC TACAAGCCGA AGGACAAGAC GTTTGAGCAG GTCCTGAAGG

3121 GCTTCGTGAC GCGCGTGCAG GCGATGGCTT GGTATACGGC GATGTCGGGC TTTTTCAACG TCTCGATTGG TGCGGGTTCC

3201 TGCCCGTCGA ATTGGGTTGT TCCGGCGACG CAGTGGAATC CCGCGTTGGA TATGACGCCG TACGTGTGCA GCAGCAGCAT

3281 GATGACGATG TACCAGCTGG GTGGGGTCGT TGTGCTGATG GTCGCGGCGT GGGCCGCGTT CAGGATTGCG TTTCTCTGAG

3361 CGGGGTGATT CATGTTTGAT GCAGTCATCA ATGCGTTGTC GGCTCTGGCG CAGTGGCTCG ACAGTGTCTT CGTCGCTATC

3441 TTCACGGCGC TTTGGCAGAT CTCAGAGGAT CTGTTCATCG ACTCGCTGGA CCTGCTATTG CAGGGCGTGA CGGCGGTCCT

3521 GAACACGTTG CCTGCGCCGA CGTTCCTGAG CGGTGTGAGC TTGCAGGCGG CGTTCAGCTC GTTGGGCGGC GACATCCTTT

3601 TTTTCTTCGG CGTCTTCAAC ATCGGACAGG GCATCGGCCT GCTGGGTGGT GCCTTCGCGT TTCGTATGGC GCGCAAGGTC

3681 GTGACGCTTT TCCAATGGTG ATGCCATGCT GATCGTTCAC GAGGGGTTGC CCGGTGCGGG CAAGACGTGG GAAGCCGTGG

3761 TCAAGCGGCT GATCCCTGCG CTACAGAAAG GCCGCAAGGT CTATGCGCGT ATCAACGGGC TGGATCACGC GAAGATCGCA

3841 GAGGTGGCCG GCGTTGAGCT GGAGCGGGTC AAGGAACTGC TGCACGAGAT TCCTGAAGCT GAGGTGCTGC GTTGGAATGA

3921 GCTGGTCGAG AACGACAGCC TGGTCATCTT GGACGAGGCG CAAAACTTCT GGCCGCATGG GGCAACGCGG ACGATGCCGC

4001 CTGATCAGAT CAAGGCGGTT GCGGAGCATC GCCACAGGGG CCTGGACATC GTGCTCATGT GTCAGGTTCT GCAAGGCGCG

4081 GGCGGCGTGC ATCCGGTGTG GGTCAACCGG GTCGATCAGA AGATCGTCTT CGAAAAGCTC AATGCGCGCG GCAAGGACGA

4161 CAAGTACAAG TGGACTGCGT ACAAGGGGCT GCACAACGGC ACGAAGATCA AGTTCACGCA GATCAACAAG GGGACTGAGG

4241 GCTACGATCC AAAGTACTTC GGCACGTATG CGAGCCATCA GGCGTCGACG GAAAACACCG AGACGTATAA GGATGCGCGG

4321 ACGAACGTTT GGAATAATCC GGTGCTCAGG CGCTGGTTGC CGCTGTTCGG CGCCGCGTTG GTGGTCGCAG TTTGGTATCT

4401 CTGGCATGCC CTGAAGGGCG GTGGCCTGGA GCAGAGCATC AATGCTGGTC ACAAGGTCGA GACGAAGACG ACAGTCGTGT

4481 CAGCGCCTGC CCCGGTAGCG CTGCCGGCGG CTGCATCTGG CGTGCAGGCT AGCGTGAGTC AGGCGCAACC GATGGCGTCG

4561 AAGGCTGAGC CTGTGAGCGG CGGTCAGAGG CAGGATGCGA TGGCTGATGA CTACGTGGCG TCGATTTCGC AGAAGTGGCG

4641 GCCGCGGCTA TCGGGGTTGG CCTGGGCCGC GGATAAGGCA CGGCTGGTGG TCGAGTGGTA CGACGAATCG AACAGGGTCA

4721 AGGAACGGCT GAGCGCGGCG CAGCTGGAGG AATTCGGCTG GGGTGTTGCG CGATCGGCCT ACGGCGATCA CGTGATCCTG

4801 TCAAAAGGCG GCGTGCATGT TGCGGTGACG AGCTGGCCGA TTGAGGAGTT CGGCCGGGTC AGCGACCGGG ATAACCGAGC

4881 GCTGCATGAG ATGGCCGTCG GCAAGGGCGA TGGTTCGTCC TCAAGCGAGC TACCCAGTTC GATGCGCGTG CCGGTTGTGC

4961 CGGTCGATGC GCGGGATGTG TCGGCGCCCG ACAGCCGGCA GAGTTGGTCG CATGGCTAGA GTTGGGCCGG CCGGCAGCTA

5041 GTGAAAAATG GTCGCGCGGA TCTCGCCGGC CATTTTTCGT CTAGCGTCAG GAGTGTCCGC GCGGATGTCG AAGCCGCTAG

5121 GGGGCCCCTT TGGGGATGTG GGAGTGTGGA TGAGGGCAGG ACGGTTGTCG CCGGAGGCGT CTGGCGGGAC CGAGTAGCGG

5201 GTAGGACTGG GGCGGTGAAG ACAGGGTGAG GGTTGGGAAG ACCTCCCTCT TGCAAGCCCC CTCTGCTGCG TTTGGCGGGC

5281 CGCTGCGCCG TTTTCCGGAG GTGGTAGGTA CGTGGGGAGG GGTGACGGTC GCGCGCCCCG CCCGCCCGCA GCGAAGCGAG

5361 GACGGACGGG GCGCGCGCAG CGCGCCCCTA CTTGTAGTAA TAACACTTAA CGGAAAGAGA GGACGGAAAG CCAAGCGCCA

5441 CAACGGTTTG CAGGCTGACC GGCCAAAGAA AAAGCCCCGC CAGCGTGCCA ACGCTTCGGG GCCGACACGG TAGCTCTAAC

5521 GGTTGGACAC CCTACCATGC GTGAAACTAC GATAGCAGAA AACTATGATT ATGGACAAGC GGCCATTTGG GGGAGTGATG

5601 CGCATCGGGC GAGCGTTGAG GTTCGCAAGA TCGAGCGTGA GGAGTCGGAT GCAGAGTTGT GGGCGGAGGG GCCGGCGCGT

5681 CATGACATGG TTGCCAAGGT TCGGGCGTTC GAAGATGGCA GTGTCGAGTT CAGCGGTTAT CGTCGTACCG TGGTGCAGCG

5761 CCTGAACGAT CTGCGTGATC TTCCCCGCCG TGCACGCGGC GCCAAGCCTG AAAATGAAGA CGATAAAGAG GCACGGGCCG

5841 CTAGCGTCAA ATCTGCTGCG AAGCGAGCTA AACAGAACGT TCGCTTGCGC TGCAAGACTG CTCGCGTCAC GCACATGATT

5921 ACGCTGACTA CGCGCGAGTG CATTACCGAT CTGGACTGGT TCCTGGGGCT CTGGGACGCT TTTCGGCGTG CGATGGCTCG

6001 CTACAGTCAA TTCCACTACA TCGCGGTTCC TGAGCTGCAG AAACGCGGCG CATGGCATAT GCACGTCGCC GTCTCAGGTC

6081 GGGTCGCGCT CAACCTCGCG CGGCGGGTAT GGCTGAAGGT CGTGGGCGGT CGTGGCAAGG GGTACTGCCA CATTCGCAAC

6161 CCTCAAGGGG CGCACTTCGG CAAGCAATGG AAGTTGGATG CGCTGGCCAG CTATGTCGCC AAGTACATCG GCAAGGACAT

6241 TGCTGAGACT CGGTTCAACA AGAAAAAGTA CTACACCAGT CGCGGCATCA ACGTTCCTGA GGCCGTGGTC TATGCGATCG

6321 AGAACAGTCG CTCAGACTGT GCGGACGCAC TGAAGGACGT GCTCACGACG CTGTGCGCCG AGTTCGATAT CGCGGACATA

6401 AGATGCTTCG TCGCCCTCGA TGGCTCGTCG TATTTCGCTA GTGCGTCTAA GGCTGTGCTG CTCGCGGCCT AGCAGCCCCG

6481 ATCGCTCATT TCCTGCTGAA CGGTCATGAT CTGTGCCCGC ACGTTGTCCT GTTCCAGTTT CCATCCCGTT CTGCGTAGCT

6561 GGTCGTTGAG CATGCTGAGG TCGACGGTTA GTCGCGCGCA TCGGGCGGCA TCGTAGGGAA CGGTCCTTGA TGTCTGCGTG

6641 GTTACAGTCC CGTCGGAGCG CGTTGTGGTC GTCGTCGTAG TCGTGACGGT ATATGCGTTC GCGGCGATGG ATACGGTTGC

6721 TGCCGATACT GCGATGAAGC TCTTCATGGC GTTCGTTTAG CGGTGGTTAT GCTGCGTCGA GTAGGCTGGG CTGCTGGCGC

6801 TCTTGGTGGC GCTTAATTGC ACGTCTGATG CTGCTGATGT GTGTGCCGAA TTGCCGTGCG ATAGCTGTCT TGGTCATCTG

6881 GCCGGCAAAC CAAAGTCGCA GTGCTTCTGC TTCGTCTTCC GGACTCAATG CGTAGTGCCT GCCCAGCTTT ACGCCTCGCT

6961 GTACGGCGGC CGCCATGCCT GCCCTGGTGC GTTCGCGGAT GAGTTCTCGT TCGAATTCGG CAAATGCCCC GACCATGTGG

7041 AAGAGCATGC GGCCGGCCGG CGTGGTGGTG TCGAGCGATT CGGTCAGCGA CCGGAATTGA GCGCCCTTCT CTTCGATCCG

7121 CTCGATGATG CGCAGCAGGT CTTTGAGTGA TCGCGCAATA CGGTCCAGCT TGTAGACGAC GACTTGGTCA CCGTGCTTGA

7201 GTATGCGGAG CATTTTCTCC AGCTCGGGTC TTCCTGCGGT TGTTCCACCG CTTTTCTTTT CGGAAAAAAT AAAACCGACG

7281 CCTGCTTTTG ACAGAGCGTC GGTTTGTGCG TGGGTCTCTT GTTCCTGCGT AGAGACCCGG GCATAGCCGT ATAGCATGCA

7361 ACCCCTCGAA AACGTTTTTT CTTGGGATTG TACTGGCTGC CCTAAGCAAT GGCGGAGAGG GTCCTTTTCG AACTCCTCCC

7441 GTGCTTAGGG CGACGGGTCA GGATGCCATC CGGACCGTCT GTTTCTTGGC CCTAAGCAGG TTCCGAAAAA CCTTCTTCAC

7521 GTTCTCGTAG ATGCCCATGC GGCGCGCCTC TTCGGCTGCG ACTGCGCATA GGGCCTGGGC GGCGCTGACG CCCGCTTCTT

7601 TCGCCAGGAT GAGTGCCGCT GCATATGTCG GCAGTCGGCT GCCCTTGACG TATCGGTCAA GGCTTGGCTG GGGGATGCCC

7681 ATCTCGTGGG CAGCTTTGTT GACCGTTCGG TCATGCAAAG CCTTATCAAT CAGTTCCGCA TATTCCATAT AAATCCTCAA

7761 TTGGTGAAGT TACCCCGAGC GGAGTAATGT CTGCATGTCA CTCCGAACGG GGTAACTCTG TTTTGAGTGA CAGGTAAACC

7841 ATACACCAAA TCGACCGCCC TACGGGGCTA CTGCCAGAAG CGGGTCGATG GTGGGTGGTC CAAGCAACCT TTCCTACCTA

7921 GCTCATTCG
